# Characteristics and Impact of HPV-Associated *p16* Expression on Head and Neck Squamous Cell Carcinoma in Thai Patients

**DOI:** 10.31557/APJCP.2020.21.6.1679

**Published:** 2020-06

**Authors:** Chuleeporn Jiarpinitnun, Noppadol Larbcharoensub, Poompis Pattaranutaporn, Teeranuch Chureemas, Jitlada Juengsamarn, Narumol Trachu, Somthawin Lukerak, Phichai Chansriwong, Nuttapong Ngamphaiboon

**Affiliations:** 1 *Division of Radiation Oncology, Department of Radiology, Faculty of Medicine Ramathibodi Hospital, Mahidol University, Bangkok, Thailand. *; 2 *Department of Pathology, Faculty of Medicine Ramathibodi Hospital, Mahidol University, Bangkok, Thailand.*; 3 *Division of Medical Oncology, Department of Medicine, Faculty of Medicine Ramathibodi Hospital, Mahidol University, Bangkok, Thailand. *; 4 *Reseach center, Faculty of Medicine Ramathibodi Hospital, Mahidol University, Bangkok, Thailand. *

**Keywords:** HPV- p16, Thailand, head and neck cancer, head and neck squamous cell carcinoma

## Abstract

**Background:**

Head and neck squamous cell carcinoma (HNSCC) is a common malignancy in Asia. Infection by human papilloma virus (HPV) has been recognized as an etiological risk for HNSCC, especially oropharyngeal region. While the association between HPV and HNSCC has been well evaluated in Western countries, only a few investigated the HPV-associated HNSCC in Southeast Asia. This study evaluated the prevalence, the characteristics, and the impact of HPV on the treatment outcomes in Thai HNSCC patients.

**Methods:**

Non-nasopharyngeal HNSCC patients treated at Ramathibodi Hospital during 2007-2013 were identified through the cancer registry database. Baseline patient, treatment data and survivals were retrospectively reviewed. The formalin-fixed paraffin-embedded (FFPE) tissue sections were retrieved for *p16* analysis. The HPV status was determined by p16 immunohistochemistry. The survival outcomes were analyzed in cases which* p16* status was confirmed.

**Results:**

Total of 200 FFPE tissues of HNSCC patients was evaluated for *p16* expression. Positive p16 status was observed in 24 cases (12%); majority of p16-positive were men (20:4 cases). The oropharynx (37.9%) was the most common site found in p16-positive while oral cavity (3.2%) was the least common site. Interestingly, 66.7% of *p16*-positive were former/current smokers, and 70.8% of this subgroup was categorized as clinical AJCC stage III-IV. The p16-positive HNSCC was significantly superior in 5-year overall survival [5-yrs OS 63% vs. 40%, p=0.03], 5-year disease-free survival [5-yrs DFS 61% vs. 36%, p=0.03] and in 5-year locoregional relapse-free survival [5-yrs LRFS 93% vs. 68%, p=0.018] when compared with *p16*-negative.

**Conclusions:**

In comparison to the results from the Western countries, the prevalence of HPV-related HNSCC in Thai patients was less, and differences in some characteristics were observed. Nevertheless, improvement in OS, DFS and LRFS were observed in *p16*-positive patients. Our analyses suggested that *p16* status is also a strong prognostic marker for HNSCC patients in Thailand.

## Introduction

Head and neck squamous cell carcinoma (HNSCC) is considered as one of the most common malignancies worldwide. Based on GLOBOCAN 2018 database, there were more than 800,000 new cases and 400,000 deaths worldwide from HNSCC (Bray et al., 2018). In United States, HNSCC approximately accounts for approximately 3% of all malignancies, with total of 53,000 new cases diagnosed and responsible for over 10,000 deaths (Siegel et al., 2019). Even higher incidences and mortality rates attributed to HNSCC were observed in Europe. In 2012, the estimated number of new HNSCC cases were 140,000 cases, accounting for 4% of all cancers in Europe, and the estimated number of deaths due to HNSCC is 63,500 (Gatta et al., 2015). Note that the geographical variation in the incidence of HNSCC is large. We are particularly interested in HNSCC because the largest burden of head and neck cancers occurs in developing countries, especially in Southeast Asia. In Asia, the estimated number of new cases was 415,000 cases with 231,000 deaths, accounting for 55% of cancer cases (Bray et al., 2018).

Traditionally, the major risk factors associated with HNSCC include smoking, alcohol consumption and betel nut chewing. Over the past decades, the decrease in oral cavity cancer incidence rate was observed due to the decline of tobacco consumption. Interestingly, the incidence of oropharyngeal cancer was elevated in economically developed countries (Chaturvedi et al., 2013). Mounting evidences from epidemiological, pathological and molecular studies have led to the implication of human papilloma virus (HPV) as an etiological factor for HNSCC, especially oropharyngeal sites (Termine et al., 2008; Mehanna et al., 2013).

HPV is a small non-enveloped virus, containing a double-stranded deoxyribonucleic acid (DNA). In human, HPV virus can cause the skin and mucous membranes infections. More than 200 types of HPV have been identified. With regards to their ability to induce cancer, HPV are categorized into 2 groups—low-risk and high-risk HPV types. HPV type 16, 18, 31, 33, 35, 39, 45, 51, 52, 56, 58, 59, 68, 73, and 82 are classified as high-risk type (Muñoz et al., 2003). Kreimer et al., (2005) conducted a systematic review of published HNSCC biopsies that HPV DNA detected. Total of 5046 specimens from 60 reported studies from 26 countries were identified. The analysis showed that HPV16 was the most common high-risk HPV type detected in all sites, particularly in the oropharynx. Of HPV-positive oropharyngeal SCC (OPSCC) cases, HPV16 accounted for 86.7%. 

HPV-associated OPSCC exhibits different clinicopathologic features when compared to those of non-HPV-associated OPSCC. HPV-associated OPSCC is likely found in non-smoker, middle-aged, white men. Patients often clinically present with advanced cervical lymph node metastases from a small size of primary tumor, which predominantly arise from tonsil or base of tongue region (Husain and Neyaz, 2017). Interestingly, recent evidence supports that HPV-associated HNSCC patients, especially those with oropharyngeal cancer, demonstrated improved survival outcome, seen as superior overall survival (OS) and disease-free survival (DFS), when compared to non-HPV-associated HNSCC patients (Ragin and Taioli, 2007; Chung et al., 2014). Therefore, HPV status could be considered as a prognostic factor. 

However, the studies of prevalence of HPV-associated HNSCC were based on populations in Western Europe, United States, and Australia (Panwar et al., 2014). The prognostic, significance, and correlations of high-risk HPV infection in HNSCC in developing countries such as Thailand cohort are lacking. Here in, we evaluated the prevalence, the characteristics, and the impact of HPV on the clinical outcomes of treatment in Thai HNSCC patient.

HPV DNA detection using either polymerase chain reaction (PCR) or in situ hybridization (ISH) approaches can be used to determine HPV status. However, the gold standard method for identifying HPV status in the tumor is to measure HPV E6/E7 mRNA transcripts. During carcinogenic progression, the viral oncoproteins, E6 and E7, are expressed in HPV-positive carcinomas. E6 and E7 oncoproteins have been shown to inactivate p53 and Rb proteins, respectively. As a result, the control of cell-cycle is disrupted, leading to impairment of cell differentiation, increased mutation, and chromosomal instability (Husain and Neyaz, 2017). 

In addition to *E6 *and *E7 *oncogenes, *p16* overexpress has been clearly linked to HPV infection. HPV-expressed *E7 *could inactivate Rb protein, leading to the transcription of the cyclin-dependent kinase inhibitor p16. Hence, p16 immunohistochemistry (IHC) has been recommended as prognostic test as *p16* expression can be presumed to be a reasonable surrogate biomarker for high-risk HPV infection in the tumor. (Shi et al., 2009) 

To evaluate the prevalence, the characteristics, and the impact of HPV on the clinical outcomes of treatment in Thai HNSCC patient, we conducted a retrospective review of baseline patient characteristics, treatment data, and survivals of Thai patients. The p16 IHC was used to determine HPV status. The survival outcomes of confirmed p16 status cases were analyzed. 

## Materials and Methods

*Patient population and tissue samples*

Non-nasopharyngeal HNSCC patients who treated at Ramathibodi Hospital between January 2007 and December 2013 were identified through the cancer registry database. We retrospectively reviewed the medical records of all patients to abstract data regarding baseline patient characteristics, treatment data, and survivals. Deaths were crosschecked with the National Security Death Index. The formalin-fixed paraffin-embedded (FFPE) samples of HNSCC tissues obtained at diagnosis were retrieved from pathology department. Patient with squamous cell carcinoma (SCC) of unknown primary, with metastatic HNSCC, and with insufficient or unavailable FFPE sample and/or incomplete or unavailable medical records were excluded. The study was approved by the ethical clearance committee on human rights to research involving human subjects, Faculty of Medicine Ramathibodi Hospital, Mahidol University.

HPV status was defined using *p16* IHC. The IHC of the expression of HPV status were characterized by CINtec® *p16* histology, consisting of a mouse monoclonal primary antibody against *p16INK4a* (Clone E6H4TM; Roche, USA). VENTANA detection kits and a VENTANA BenchMark XT automated slide stainer were used to detect *p16* expression from archived FFPEs. According to the manufacturer’s instructions, the *p16* expression was categorized as positive or negative; positive *p16 *expression was defined by tumor cells demonstrated nuclear and cytoplasmic staining ≥70% of tumor cells.

*Statistical analysis*

Statistical analyses were performed using Statistical Package for Social Sciences for Windows version 17.0 (SPSS V.17.0). Descriptive statistics were used to describe patient characteristics, details of treatment, and prevalence of p16 status. The categorical variables were described in frequency (percentage) and the continuous variables were shown in median (range). Fisher’s exact and Chi-square test were used to compare between groups, which divided by p16 status. Survival analyses were calculated using Kaplan-Meier method and the comparisons between groups were evaluated by log-rank test. Multivariate survival analysis was computed using Cox proportional hazard regression model. The survival time was defined starting from the date of pathological confirmed diagnosis to date of first event or last follow-up. The event of overall survival (OS) was defined as time from diagnosis to death from any cause. Either death or the date of any first recurrence was the endpoint of disease-free survival (DFS) while the first local and/or regional recurrence was the endpoint of locoregional relapse-free survival (LRFS). A p-value of <0.05 was considered to indicate statistically significance.

## Results

*Patient and treatment characteristics*

**Figure 1:**
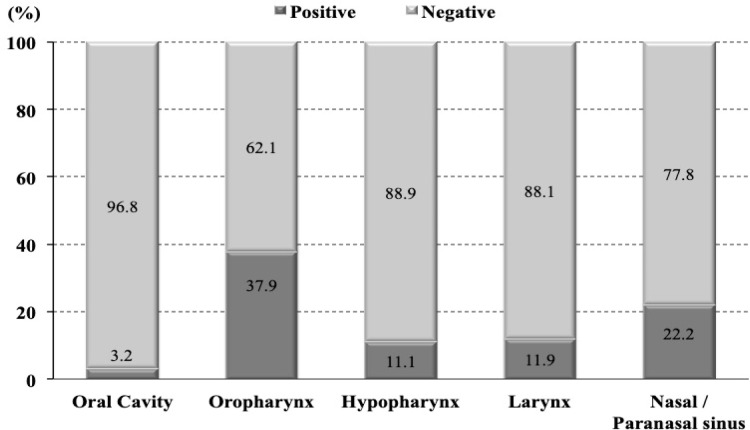
Distribution of p16 in Head and Neck Region

We identified 200 patients diagnosed with non-nasopharyngeal HNSCC and received treatment during 2007-2013 at Ramathibodi Hospital, Thailand. Baseline patient, tumor, and treatment characteristics were summarized in Table 1. FFPE tissues of all identified HNSCC patients were analyzed using the status of HPV by p16 IHC. The p16 status was positive in 24 of 200 cases (12%). Majority of p16-positive patients were men (20:4 cases). The oropharynx was the most common primary site with the distribution of p16-positive status of 37.9%, followed by nasal/paranasal sinus (22.2%), larynx (11.9%), hypopharynx (11.1%), and oral cavity (3.2%). The distribution of p16-positive expression in the head and neck region was shown in Figure 1. In p16-positive group, 66.7% were former/current smokers while 20.8% were non-smokers. Approximately 70% of patients with p16 positively expressed were presented with locally advanced stage (stage III/IVa-b). However, the baseline patient and pathological characteristics were not statistically different between p16-positive and p16-negative groups except the site of primary tumor (p<0.001) and the degree of tumor differentiation (p=0.007). 

*Overall treatment outcomes*

**Figure 2:**
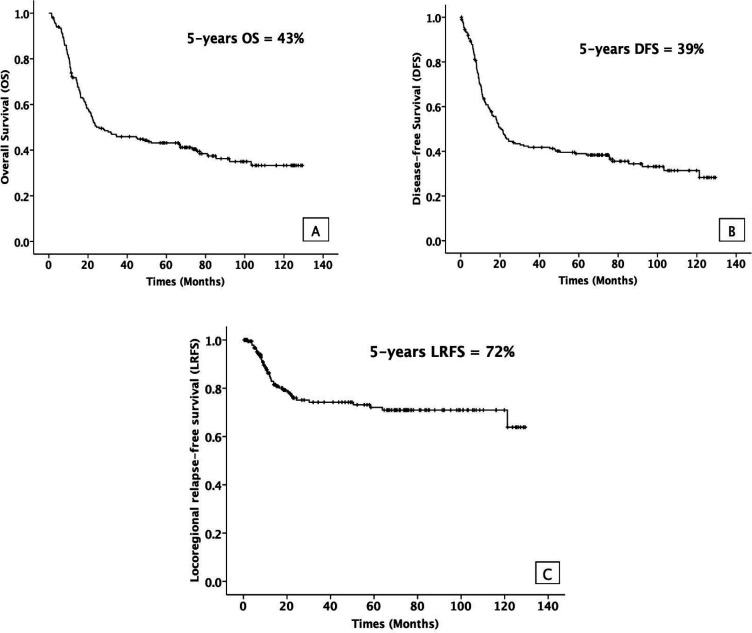
5-Year Survival Rates for (A) Overall (B) Disease-free (C) Locoregional relapse-free

Kaplan-Meier survival curves displaying treatment outcomes for all 200 non-nasopharyngeal HNSCC patients were illustrated in Figure 2. The median follow-up time was approximately 38.5 months (ranged from 1 to 129 months). At the last follow-up, 79 patients (39.5%) were alive even though 5 out of 79 patients were alive with disease. Disease recurrence occurred in 60 patients. The most common type of recurrence was locoregional recurrence, which was found in 43 patients. However, 8 of 43 patients had both locoregional relapse and distant metastasis. Lung was the most common site of distant metastasis. The 5-year OS was 43% while the 5-year DFS and 5-year LRFS were 39% and 72%, respectively. 

*P16 status and treatment outcomes*

**Figure 3:**
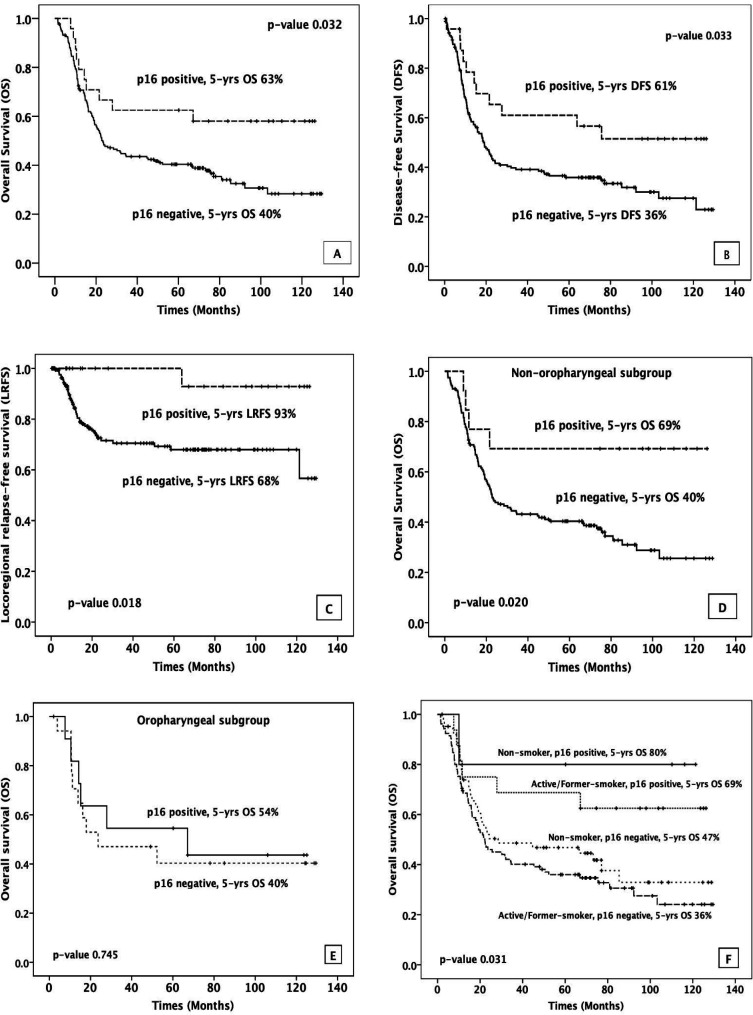
5-Years Survival Rates for (A) Overall (B) Disease-free (C) Locoregional relapse-free between p16-positive and p16-negative in all HNSCC patients, and 5-years OS between p16-positive and p16-negative in (D) non-oropharyngeal subgroup (E) oropharyngeal subgroup, and (F) 5-years OS of the combination between p16 status and smoking status

At the last follow-up, 14 patients (58.3%) with p16-positive status were alive, of which one patient was alive with distant metastasis. Patients with p16-positive showed lower disease progression when compared to p16-negative cohort (11:114), and a similar trend was observed in loco-regional recurrence (1:42). In comparison to p16-negative HNSCC patients, significantly superior 5-year OS, 5-year DFS, and 5-year LRFS outcomes were seen in p16-positive patients (5-year OS 63% vs 40%, p=0.032, 5-year DFS 61% vs 36%, p=0.033, and 5-year LRFS 93% vs 68%, p=0.018, Figure 3A-C). Moreover, the subgroup of 13 patients with non-oropharyngeal p16-positive also presented statistically significantly better treatment outcome of 5-year OS (69% vs. 40%, p=0.020) (Figure 3D), 5-year DFS (69% vs. 36%, p=0.033) and 5-year LRFS (100% vs. 68%, p=0.043). However, we did not observe statistically significant improvement in survival outcome when comparing 11 patients of p16-positive in oropharyngeal subgroup to patients with p16-negative status (5-year OS 54% vs 40%, p=0.745 (Figure 3E), 5-year DFS 50% vs 35%, p=0.538 and 5-year LRFS 80% vs 67%, p=0.213).

*P16 status and smoking*

Among 24 patients with p16-positive status, 16 patients were active or former smoker and 5 patients were non-smoker. The p16-positive patients with active or former smoking status had worse treatment outcome compared to p16 positive non-smokers (HR 1.886, 95%CI 0.227-15.679; p=0.557) with 5-year survival decreased from 80% to 69%. Moreover, when we focused the analysis on smoker subgroups (121 patients), the patients with p16-positive status showed statistically significantly better 5-year OS than patients with p16-negative status (69% vs 36%, p=0.021). Moreover, this positive effect of p16 overexpression was also observed when we analyzed the non-smoker subgroups (5-year OS 80% vs 47%, p=0.132). Based on our data, the active or former smoker with no p16 overexpression was the group of HNSCC patients that had the worst treatment outcome (Figure 3F).

*Factors affecting treatment outcomes*

Based on the univariate analysis, three prognostic factors showed statistically significant impact on OS and DFS. These factors are p16 status (positive vs. negative), AJCC7th staging (stage I-II vs III-IV), and ECOG performance status (ECOG 0-1 vs. ≥2). The status of p16 expression and AJCC staging also had significant impact on LRFS. However, as shown in Table 2, ECOG performance status did not show association with LRFS. Similar results were observed in multivariate analysis. The p16-positive status was identified as a good prognostic factor influencing OS (HR 0.435, 95%CI 0.214-0.842, p=0.014), DFS (HR 0.443, 95%CI 0.231-0.850, p=0.014) and LRFS (HR 0.125, 95%CI 0.017-0.913, p=0.040). On the contrary, patients with AJCC7th stage III-IV showed poor prognosis for OS (HR 3.113, 95%CI 1.935-5.009, p<0.001), DFS (HR 3.060, 95%CI 1.922-4.874, p<0.001) and LRFS (HR 2.039, 95%CI 1.001-4.153, p=0.050). 

**Table 1:** Baseline Patient Characteristics and p16 Status

	P16 status		*P* value
	Positive (n=24)	Negative (n=176)	
Sex			
Male	20 (83.3%)	125 (71.0%)	0.234
Female	4 (16.7%)	51 (29.0%)	
Age (years)			
<65	11 (45.8%)	95 (54.0%)	0.453
≥ 65	13 (54.2%)	81 (46.0%)	
Median (range)	65 (41-84)	63 (25-89)	0.717
ECOG			
0-1	20 (83.3%)	144 (81.8%)	0.772
≥ 2	3 (12.5%)	30 (17.0%)	
Smoking			
Never	5 (20.8%)	63 (35.8%)	0.218
Active/Former	16 (66.7%)	105 (59.7%)	
Median pack-year (range)	20 (0-60)	10 (0-150)	0.384
Primary site			
Oral cavity	3 (12.5%)	90 (51.1%)	<0.001
Oropharynx	11 (45.8%)	18 (10.2%)	
Hypopharynx	3 (12.5%)	24 (13.6%)	
Larynx	5 (20.8%)	37 (21.0%)	
Nasal and Paranasal sinus	2 (8.4%)	7 (4.0%)	
Tumor differentiation			
Well	1 (4.2%)	57 (32.4%)	0.007
Moderate	10 (41.7%)	55 (31.2%)	
Poorly/Undifferentiated	8 (33.3%)	29 (16.5%)	
Tumor stage			
1-2	18 (75.0%)	78 (44.3%)	0.005
3-4	6 (25.0%)	98 (55.7%)	
Nodal stage			
0-1	16 (66.7%)	123 (69.9%)	0.748
≥ 2	8 (33.3%)	53 (30.1%)	
The AJCC 7th stage			
I-II	7 (29.2%)	50 (28.4%)	0.939
III-IV	17 (70.8%)	126 (71.6%)	
Definitive treatment			
Surgery	9 (37.5%)	103 (61.7%)	0.078
Chemoradiation	10 (41.7%)	33 (19.8%)	
Radiation alone	1 (4.2%)	9 (5.4%)	
Unknown	4 (16.7%)	22 (13.2%)	
Adjuvant treatment			
No Adjuvant	3 (12.5%)	38 (21.6%)	0.945
Chemoradiation	3 (12.5%)	29 (16.5%)	
Radiation alone	3 (12.5%)	36 (20.5%)	

**Table 2:** Univariate and Multivariate analysis for OS, DFS, and LRFS

Factor	Univariated		Multivariated		Univariated		Multivariated		Univariated		Multivariated	
	HR (95%CI)	*P*-value	Adjusted HR (95%CI)	*P*-value	HR (95%CI)	*P*-value	Adjusted HR (95%CI)	*P*-value	HR (95%CI)	*P*-value	Adjusted HR (95%CI)	*P*-value
P16 Status												
Positive vs Negative	0.498 (0.260-0.954)	0.036	0.435 (0.214-0.842)	0.014	0.515 (0.276-0.959)	0.036	0.443 (0.231-0.850)	0.014	0.13 (0.018-0.950)	0.044	0.125 (0.017-0.913)	0.04
Sex												
Female vs Male	0.731 (0.481-1.110)	0.142			0.686 (0.452-1.039)	0.075			1.019 (0.531-1.955)	0.956		
ECOG												
≥2 vs 0-1	1.973 (1.289-3.019)	0.002	2.261 (1.471-3.475)	<0.001	1.817 (1.196-2.762)	0.005	2.043 (1.340-3.115)	0.001	1.339 (0.618-2.903)	0.46		
Smoking status												
Former/Active vs Never	1.226 (0.828-1.815)	0.309			1.183 (0.806-1.736)	0.391			0.78 (0.416-1.459)	0.437		
Age (years)												
≥ 65 vs < 65	1.164 (0.815-1.664)	0.404			1.088 (0.765-1.545)	0.64			0.892 (0.488-1.630)	0.71		
Tumor site												
Non-oropharynx vs Oropharynx	1.203 (0.710-2.038)	0.492			1.11 (0.672-1.833)	0.685			1.197 (0.501-2.860)	0.686		
The AJCC 7th Stage												
I II-IV vs I-II	2.985 (1.861-4.787)	<0.001	3.113 (1.935-5.009)	<0.001	2.912 (1.833-4.624)	<0.001	3.06 (1.922-4.874)	<0.001	1.971 (0.968-4.015)	0.061	2.039 (1.001-4.153)	0.05

Value presented as hazard ratio (95% confident interval), p-value corresponds to Cox regression analysis

## Discussion

This present study investigated the prevalence, characteristics and impact of HPV, which detected by p16 immunohistochemistry, on clinical outcome of treatment in Thai HNSCC patients from a single tertiary referral hospital in Thailand. As the data of the association between HPV and HNSCC in Southeast Asian counties are lacking, this study could provide the impact of HPV-associated *p16*-expression in HNSCC patients in this region.

An increase in prevalence of HPV-associated OPSCC has been reported over past decades, especially in Western countries. The mean prevalence of HPV association with OPSCC was considerably high, up to 50-70% in Europe and North America (Stein et al., 2015). However, data from Asia-Pacific countries indicated that the prevalence of HPV-associated OPSCC was lower than that in the Western countries. In Asia-Pacific region, the overall HPV prevalence was approximately 40% (10-50%) (Shaikh et al., 2015). To the best of our knowledge, there was only one recently published report on HPV prevalence of HNSCC in Thailand, available in the international database. Nopmaneepaisarn et al., (2019) reported positive *p16*-expression in 28.2% of OPSCC; however, only 14.5% of OPSCC associated with a high-risk HPV when confirmed by ISH. In comparison to our study, the prevalence of HPV related OPSCC in our study is significantly higher with 38% of OPSCC associated with p16-positive status. The difference in prevalence of HPV-associated OPSCC might be due to the variation in methods used to detect high-risk HPV. In our study,* p16* (INK4A) IHC was employed as the method presents many benefits in its simplicity, cost-effective, reproducible. In addition, *p16* (INK4A) IHC has been used in many clinical trials and has recommended by Fakhry et al., (2018) as a surrogate marker for HPV status for OPSCC. When compared to Nopmaneepaisarn (2019), the difference was that we did not use PCR or ISH to further validate the p16 immunohistochemistry results, and that might lead to the difference in HPV prevalence rate observed. With regards to the non-oropharyngeal squamous cell carcinoma (non-OPSCC) cohorts, lower prevalence of p16-positive was shown in our study. We found p16 overexpression in only 3-11% of non-OPSCC, which is consistent with the previously reported data from Nopmaneepaisarn (2019) conducted in another tertiary referral center in Thailand. However, in Nopmaneepaisarn et al., (2019) study, when validated these p16-positive non-OPSCC cases with HPV DNA ISH, it was found that prevalence of HPV associated non-OPSCC was decreased from 3-4% to 1.5%. The low concordance between p16 overexpression and the presence of HPV in non-OPSCC were also reported in previous western published data (Chernock et al., 2013). Hence, unlike oropharyngeal site, to employ p16 immunohistochemistry as a surrogate for determining HPV status in non-OP site such as the oral cavity, larynx and hypopharynx, further studies may be needed.

HPV-associated HNSCC had unique clinical and demographic features when compared to non-HPV-associated HNSCC. HPV-associated HNSCC mostly found in patients with male gender, younger age, and less likely consume excessive tobacco. Additionally, the typical clinical presentation of HPV-associated HNSCC are small and poorly differentiated tumor with a higher incidence of advanced lymph node metastases (Spence et al., 2016). In comparison to the results from our study, similar clinical and demographic features were observed with an only difference in smoking consumption aspect. More than 65% of HPV-associated HNSCC in our study presented in whom previously or currently smoke tobacco. However, the difference in this feature is not unexpected. Sinha et al., (2012) reviewed the association between smoking and HPV in HNSCC and found that HPV-associated tumors should not be considered as an occurrence exclusive to non-smokers. In addition, the previous studies also showed an additive or synergistic interaction between smoking and HPV. Smoking tobacco could add risk to the development of head and neck cancer in whom with HPV infection. Therefore, it is important to promote smoking cessation in smokers with infected HPV.

With regards to therapeutic perspective, our study showed significant impact of p16 expression on treatment outcomes of overall HNSCC cohort of Thai patients. Patient with p16-positive status had superior OS, DFS and LRFS over p16-negative HNSCC patients. This impact of p16 status was not dependent on the other prognostic factors, such as performance status or tumor staging, that shown statistically significant prognostic factor on multivariate analysis. Mounting evidences verified the significance of p16 overexpression as a biomarker for prediction of prognosis in OPSCC patients (Ang et al., 2010; Rischin et al., 2010; Meng et al., 2018). According to our study, when we analyzed OPSCC subgroup (29 patients) in our HNSCC cohort, the results showed superior treatment outcome of OS (HR 0.845, 95%CI 0.307-2.330; p=0.745), DFS (HR 0.735, 95%CI 0.275-1.966; p=0.539), and LRFS (HR 0.278, 95%CI 0.032-2.392; p=0.244) of p16-positive over p16-negative OPSCC patients. However, the positive treatment outcome was not statistically significant, which might be due to small sample size of tumor locating in oropharyngeal site. With regards to the non-OPSCC subgroup, many studies have investigated the *p16* expression as a biomarker for predicting prognosis; however, the impact on treatment outcome in this particular group is still inconclusive and ongoing debate. Study by Lassen et al., (2014) indicated that in 479 non-OPSCC patients from DAHANCA-trials, which were treated by chemo-RT, HPV-associated p16 expression did not have a prognostic impact on the treatment outcome. The similar results were also observed in the findings by Fakhry et al., (2017). On the contrary, Chung et al., (2014) reported that non-OPSCC patients from RTOG 0129, 0234 and 0522 studies with p16-negative status had worse treatment outcomes than patients with p16-positive status. Hazard ratios for p16 expression were 0.63 (95% CI 0.42-0.95; p=0.03) and 0.56 (95% CI 0.35-0.89; p=0.01) for progression-free survival (PFS) and OS, respectively. In addition, similar prognostic role of p16 for OS (HR 0.41, 95% CI 0.25-0.69; p<0.001) in 387 non-OPSCC patients was also observed in HNSCC cohort using the US Veterans Affairs database (Bryant et al., 2018). Disparity between studies with regards to the impact of *p16 *expression on treatment outcome in non-OPSCC is not fully known. Several factors including low incidence of p16, misclassification of primary anatomical sites, and ectopic tonsillar tissue were hypothesized to be the root of discrepant results of investigations. The results of our study are in support of p16 expression as a strong prognostic factor, affecting treatment outcome for non-OPSCC subgroup patients. Non-OPSCC patients with p16-positive status have a better chance to survive longer than patients with *p-16* negative status; the hazard ratio was 0.312 (95% CI 0.113-0.858; p=0.024). However, this positive impact of *p16* overexpression on survival may not confer HPV as a prognostic factor on non-OPSCC due to the high disconcordance of* p16* expression and high-risk of HPV infection. In this present study, the limitation was that the HPV status was determined using p16 immunohistochemistry without further validated by PCR or ISH. The investigations of concordance between *p16*-expression and high-risk HPV infection and the impact of oncogenic HPV infection on treatment outcome of Thai HNSCC cohort, especially non-OPSCC subsites, are on-going and will be reported in due course.

In conclusion, we found that the prevalence of HPV associated p16 expression HNSCC in Thai patients was less than that from the data of the Western countries. Nevertheless, our findings support p16 expression as a strongly prognostic biomarker for HNSCC, especially in non-OPSCC subgroups. We strongly suggest that p16 IHC testing should be advised in the overall HNSCC population with additional confirmatory molecular testing such as PCR or ISH to be recommended for high-risk HPV in non-OPSCC subgroups. 
